# Catalytic Inhibitors of Topoisomerase II Differently Modulate the Toxicity of Anthracyclines in Cardiac and Cancer Cells

**DOI:** 10.1371/journal.pone.0076676

**Published:** 2013-10-07

**Authors:** Anna Vavrova, Hana Jansova, Eliska Mackova, Miloslav Machacek, Pavlina Haskova, Lucie Tichotova, Martin Sterba, Tomas Simunek

**Affiliations:** 1 Department of Biochemical Sciences, Charles University in Prague, Faculty of Pharmacy in Hradec Kralove, Hradec Kralove, Czech Republic; 2 Department of Pharmacology, Charles University in Prague, Faculty of Medicine in Hradec Kralove, Hradec Kralove, Czech Republic; UAE University, Faculty of Medicine & Health Sciences, United Arab Emirates

## Abstract

Anthracyclines (such as doxorubicin or daunorubicin) are among the most effective anticancer drugs, but their usefulness is hampered by the risk of irreversible cardiotoxicity. Dexrazoxane (ICRF-187) is the only clinically approved cardioprotective agent against anthracycline cardiotoxicity. Its activity has traditionally been attributed to the iron-chelating effects of its metabolite with subsequent protection from oxidative stress. However, dexrazoxane is also a catalytic inhibitor of topoisomerase II (TOP2). Therefore, we examined whether dexrazoxane and two other TOP2 catalytic inhibitors, namely sobuzoxane (MST-16) and merbarone, protect cardiomyocytes from anthracycline toxicity and assessed their effects on anthracycline antineoplastic efficacy. Dexrazoxane and two other TOP2 inhibitors protected isolated neonatal rat cardiomyocytes against toxicity induced by both doxorubicin and daunorubicin. However, none of the TOP2 inhibitors significantly protected cardiomyocytes in a model of hydrogen peroxide-induced oxidative injury. In contrast, the catalytic inhibitors did not compromise the antiproliferative effects of the anthracyclines in the HL-60 leukemic cell line; instead, synergistic interactions were mostly observed. Additionally, anthracycline-induced caspase activation was differentially modulated by the TOP2 inhibitors in cardiac and cancer cells. Whereas dexrazoxane was upon hydrolysis able to significantly chelate intracellular labile iron ions, no such effect was noted for either sobuzoxane or merbarone. In conclusion, our data indicate that dexrazoxane may protect cardiomyocytes *via* its catalytic TOP2 inhibitory activity rather than iron-chelation activity. The differential expression and/or regulation of TOP2 isoforms in cardiac and cancer cells by catalytic inhibitors may be responsible for the selective modulation of anthracycline action observed.

## Introduction

**Figure 1:**
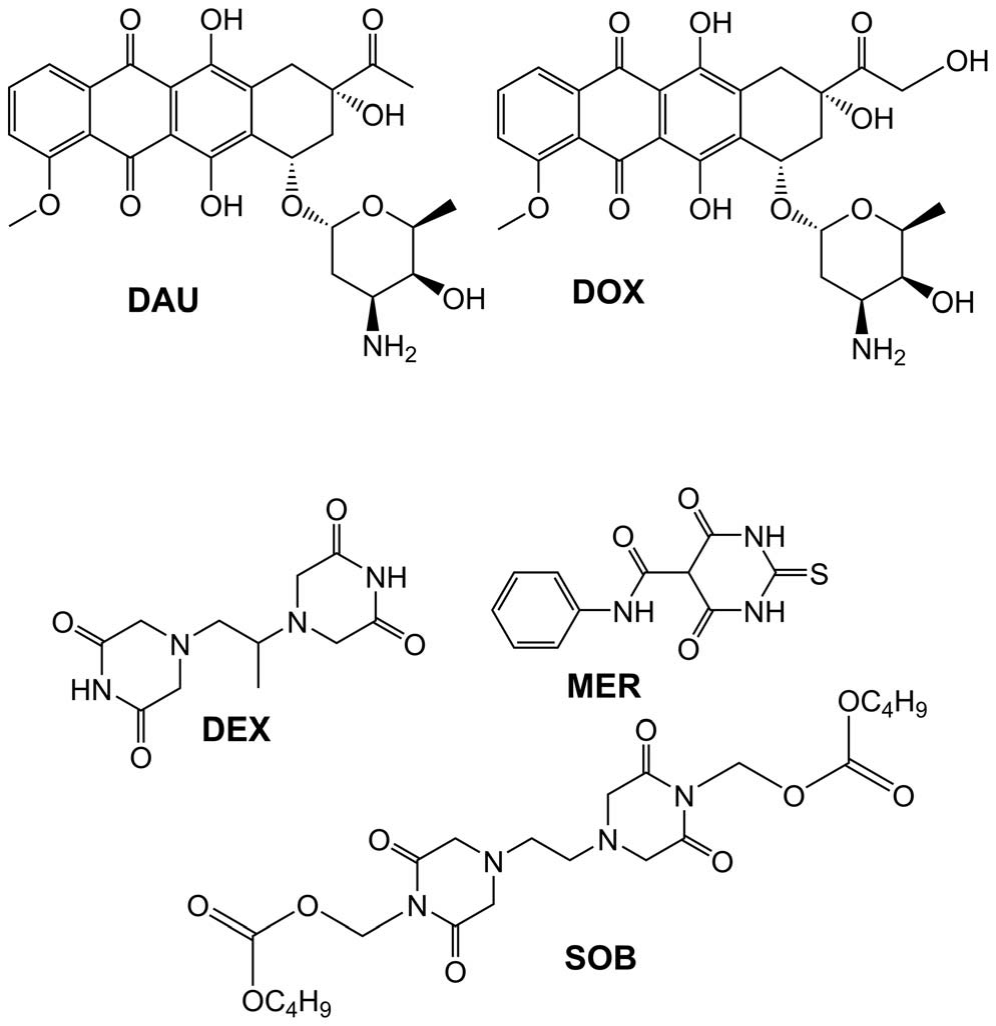
Chemical structures of the anthracyclines and the topoisomerase II catalytic inhibitors used in this study. Anthracyclines doxorubicin (DOX) and daunorubicin (DAU) and topoisomerase II catalytic inhibitors dexrazoxane (DEX), sobuzoxane (SOB) and merbarone (MER) were used in this study.

Anthracycline (ANT) antibiotics, such as doxorubicin (DOX, Figure 1), daunorubicin (DAU, Figure 1) or epirubicin, rank among the most effective and frequently used antineoplastic agents and remain indispensable components of modern chemotherapy protocols for numerous haematological malignancies as well as solid tumours. However, the risk of irreversible and potentially fatal toxicity to cardiac tissue is the main drawback of ANT use in clinical practice [1].

Numerous hypotheses have been proposed regarding the mechanisms underlying both the antineoplastic and cardiotoxic effects of ANTs [2]. Currently, topoisomerase II (TOP2) is generally recognised as the principal molecular target for ANT antitumor action. ANTs belong to the group of "TOP2 poisons", which consist of cytotoxic agents that stabilise the “cleavable complex” [3]. In terms of cardiotoxicity, the iron (Fe)-catalysed intramyocardial production of reactive oxygen species (ROS) has traditionally been implicated. The C ring of ANT aglycone readily undergoes redox-cycling, and Fe ions may form redox-active complexes with ANTs, resulting in the formation of superoxide, peroxide and eventually highly reactive and toxic hydroxyl radicals [4], [5].

This traditional “ROS and Fe” hypothesis of ANT-induced cardiotoxicity has been reinforced by the protective efficiency of dexrazoxane (DEX, ICRF-187, Figure 1), which is the only clinically approved cardioprotectant. The cardioprotective effects of DEX have been attributed to its hydrolysis product ADR-925, strikingly similar to the well-known metal chelator EDTA. Following the metabolism of DEX into ADR-925, this product can chelate free and redox-active intracellular Fe and/or replace Fe in ANT-Fe complexes, thus preventing site-specific hydroxyl radical formation and oxidative damage to cardiac tissue [6].

However, DEX is also an established catalytic inhibitor of TOP2 [7], and therefore, it cannot be ruled out that DEX may exert protective effects through interference with ANT-induced TOP2 poisoning in the heart [8], [9]. Indeed, a recent study reported that deletion of the TOP2 beta isoform (*Top2b* gene) protected cardiomyocytes from DNA double-strand breaks and transcriptome changes induced by acute in vivo DOX treatment, with subsequent prevention of defective mitochondrial biogenesis and ROS formation. Furthermore, cardiomyocyte-specific deletion of the *Top2b* gene protected mice from the development of progressive heart failure induced by repeated DOX treatment, suggesting that DOX-induced cardiotoxicity is primarily mediated by cardiomyocyte TOP2B [10].

Questions arising from these previous studies encouraged us to investigate the involvement of TOP2 in ANT cardiotoxicity and to assess other TOP2 catalytic inhibitors as potential cardioprotectants. In this study, using primary cultures of isolated rat neonatal ventricular cardiomyocytes (NVCMs), we examined the protective effects of DEX and two other catalytic inhibitors of TOP2, sobuzoxane (SOB, MST-16, Figure 1) and merbarone (MER, Figure 1), against cardiotoxicity induced by DAU and DOX. For comparison, we also investigated the effects of these agents in a model of H_2_O_2_-induced oxidative cardiomyocyte injury. Additionally, the HL-60 leukemic cell line was used to assess whether TOP2 catalytic inhibitors can affect ANT cardiotoxicity without compromising their antiproliferative efficacy against leukemic cancer cells.

## Materials and Methods

### 1. Materials

Dulbecco’s modified Eagle’s medium (DMEM), DMEM with nutrient mixture F-12 (DMEM/F12), horse serum (HS), foetal bovine serum (FBS), penicillin/streptomycin solution (5000 U/ml; P/S) and sodium pyruvate solution (100 mM; PYR) were purchased from Lonza (Belgium). The sera were heat-inactivated prior to use. DEX was obtained from Huaren Chemicals (Chang-Zhou, China). RPMI-1640 medium with L-glutamine and NaHCO_3_, lactic acid, nicotinamide adenine dinucleotide (NAD^+^), MTT (3-(4,5-dimethylthiazol-2-yl)-2,5-diphenyltetrazolium bromide, SOB, MER, dimethyl sulfoxide and 4-(2-hydroxyethyl)-1- piperazineethanesulphonic acid (HEPES) were purchased from Sigma (Czech Republic) and other chemicals (e.g., constituents of various buffers) from Penta (Czech Republic) and were of the highest available pharmaceutical or analytical grade. Dimethyl sulfoxide was used to dissolve DEX, SOB and MER. Plastic for the cell culture was obtained from TPP (Switzerland).

### 2. Cardiomyocyte isolation and toxicity assessments

All animal procedures and the preparation of NVCMs were approved and supervised by the Charles University Animal Care Committee. Primary cultures of NVCMs were prepared from 2-day-old Wistar rats. The animals were anaesthetized with CO_2_, and decapitated. The chests were opened and the hearts were collected in an ice-cold Ca^2+^-free buffer, containing 116 mM NaCl, 5.3 mM KCl, 1.2 mM MgSO_4_, 1.13 mM NaH_2_PO_4_, 5 mM glucose and 20 mM HEPES (pH 7.40). The ventricles were thoroughly minced and serially digested with a mixture of collagenase (0.25 mg/ml; Invitrogen, USA) and pancreatin (0.4 mg/ml; Sigma) solution at 37 °C. The cell suspension was placed on a large (15 cm) Petri dish (approx. 20 hearts per dish) and left for 2 h at 37°C to separate the myocytes (floating in the medium) from fibroblasts (attached to the dish). The myocyte-rich suspension was collected and viable cells were counted using Trypan blue exclusion. The isolation procedure resulted in a confluent cellular monolayer with ∼90% of cardiomyocytes beating synchronically. To assess cytotoxicity, cellular morphology and caspase activity, the cells were plated on 12-well plates pre-coated with 1% gelatine at a density of 0.8 million cells per well. To measure glutathione content, the cells were plated on 60-mm Petri dishes at a density of 4.8 million cells per dish. NVCMs were cultured at 37 °C and 5% CO_2_ in the DMEM/F12 supplemented with 10% HS, 5% FBS, 4% PYR and 1% P/S. Newly isolated NVCMs were left for 40 h, then the medium was changed to DMEM/F12 supplemented with 5% FBS, 4% PYR and 1% P/S. The medium was replaced once more after another 24 h. All experiments were started on the fourth day after isolation. Using both serum and PYR-free medium, NVCMs were incubated at 37 °C with the tested agents either alone or in combination. The activity of lactate dehydrogenase (LDH) released from cardiomyocytes was determined in cell culture media as a standard marker of cytotoxicity and cellular breakdown using a well-established spectrophotometric method; in our previous studies, this method correlated well with release of cardio-specific troponins T and I [11]. The activity of LDH released from cardiomyocytes was expressed as the percentage of total cellular LDH as measured following cell lysis for 15 min (0.1 M potassium phosphate, 1% Triton X-100, 1 mM DTT [(-) -1,4-dithio-L-threitol], 2 mM EDTA, pH 7.8) at room temperature. All the samples were frozen immediately and kept in −80°C prior to measurement. Activity of LDH was assayed in Tris–HCl buffer (100 mM, pH 8.9) containing 35 mM of lactic acid and 5 mM of NAD^+^. The rate of NAD^+^ reduction was monitored spectrophotometrically at 340 nm for 2 min. The slope of the linear region and molar absorption coefficient e  =  6.22*10^3 ^M/cm was used for calculation of LDH activity and the data was expressed as the percentage of total LDH amount.

Changes in cellular morphology were documented using an Eclipse TS100 inverted epifluorescence microscope (Nikon, Japan), and the NIS-Elements AR 2.20 software (Laboratory Imaging, Czech Republic). To visualise active mitochondria, cells were loaded with 0.5 µM JC-1 (Molecular Probes/Invitrogen, U.S.A.) for 30 min at 37°C. At low concentrations, JC-1 exists within cells in a green-fluorescent monomeric form and accumulates in actively respiring mitochondria. There JC-1 monomers aggregate driven by existing membrane potential difference (ΔΨ_m_) between the inner and outer mitochondrial membrane. This ΔΨ_m_-dependent formation of “J-aggregates” is represented by a stroke-shift from green to red fluorescence.

### 3. Proliferation studies

The HL-60 cell line, derived from a patient with acute promyelocytic leukaemia [12], was purchased from the American Type Culture Collection (Manassas, VA, U.S.A.). Cells were cultured in RPMI 1640 medium supplemented with 10% FBS and 1% P/S in 75-cm^2^ tissue culture flasks) at 37°C in a humidified atmosphere of 5% CO_2_. For proliferation assays, cells were plated on 96-well plates at a density of 10,000 cells per well. The combination studies were designed according to the Chou – Talalay method [13]. Briefly, the concentrations of chelators inducing 50% proliferation decrease (IC_50_) of all single substances were first determined. Then, both the individual substances and combination mixtures were tested at concentrations corresponding to several fractions and multiples (1/8; 1/4; 1/2; 1; 2; 4) of their individual IC_50_ values.

Cellular proliferation was assessed using a viability assay based on the ability of active mitochondria to change yellow 3-(4,5-dimethylthiazol-2-yl)-2,5-difenyltetrazolium bromide tetrazole (MTT; Sigma) to purple formazan according to the manufacturer’s instructions Briefly, 25 µl of 3 mg/ml MTT solution in phosphate buffered saline were added to the culture medium (100 µl) and after 2 h of incubation in 37°C the cells were lysed with lysis buffer (isopropanol, 0.1 M HCl, 10% Triton X-100) for 30 min in room temperature. After dissolving, the optical density of soluble MTT was measured using a Tecan Infinite 200 M plate reader at λ  =  570 nm, subtracting the λ  =  690 nm background. The proliferation rates of the experimental groups were expressed as percentages of the untreated controls (100%).

### 4. Assessment of caspase activity

The activities of caspases 3/7, 8 and 9 were determined using a commercially available kit (Caspase Glo Assays, Promega, U.S.A.) based on the ability of caspases to cleave specific amino acid sequences present in a substrate for luciferase. Caspases’ activities were determined in relation to protein content, which was assessed using the bicinchoninic acid method according to the manufacturer's protocol (Sigma). Lysates were diluted to equal protein concentrations.

### 5. Determination of total and oxidised glutathione

NVCMs were washed with PBS (2×2.5 ml, 4°C), harvested with a cell scraper, and centrifuged (10 min at 700 × g, 4°C), and the pellet was resuspended in 250 µl of 4°C cold sulphosalicylic acid (Sigma) and then sonicated on ice for 10 s (Bandelin Sonoplus, Bandelin Instruments, Germany). The samples were then centrifuged for 15 min at 18,000 × g, and the supernatants were used to determine the amount of reduced and oxidised glutathione (GSH/GSSG) according to Tietze [14] using the enzymatic recycling method with 5,5'-Dithio-Bis (2-Nitrobenzoic Acid) (Sigma) and GSH reductase (Sigma) in a microplate format. The rate of 2-nitro-5-mercaptobenzoic acid formation was recorded at 405 nm for 2 min and the slope was compared to that of the standard curve of oxidized glutathione (GSSG). The concentration of total glutathione (GSH+GSSG) was calculated using the method of linear regression, the results were corrected for protein content and expressed as a percentage of the total glutathione in a control sample (100%). GSSG content in the sample was determined after derivatization of GSH with 2-vinylpyridine (Sigma). The results were corrected for the protein content determined spectrophotometrically using bicinchoninic acid method according to the manufacturer’s protocol (Sigma).

### 6. Flow cytometry cell cycle analysis

After incubation, HL60 cells were centrifuged at 300 × g, washed in PBS with 5% FBS (PBS + FBS) and suspended in a small amount of PBS + FBS. Then, ice-cold 70% ethanol was added drop-wise, and the cells were fixed for 3 h at - 20 °C. After fixation, ethanol was removed by centrifugation; cells were washed in PBS + FBS and suspended in 4 mM sodium citrate in PBS + FBS. Finally, cells were incubated with 200 µg/ml RNAse A (Sigma) and 30 µg/ml propidium iodide (PI) for 20 min at 37 °C. Cells were analysed using an Accuri C6 flow cytometer (Accuri Cytometers Europe Ltd., U.K.) as described previously [15]. PI was excited at 488 nm, and fluorescence was analysed at 585 nm (FL-2). Per analysis, 10,000 events were collected. Cell cycle analysis was evaluated using MultiCycle AV Software (Phoenix Flow Systems, U.S.A.). Cell cycle figures were created with Cyflogic software (CyFlo Ltd, Finland).

### 7. Calcein-AM assay for determinations of intracellular Fe-chelating properties

The experiments analyzing the Fe-chelating intracellular efficiencies of the examined agents were performed according to Glickstein et al. [16] with minor modifications. The H9c2 cell line derived from embryonic rat heart tissue [17] was purchased from the American Type Culture Collection (U.S.A.). Cells were cultured in DMEM supplemented with 10% FBS, 1% P/S and 10 mM HEPES in 75 cm^2^ tissue culture flasks at 37°C in a humidified atmosphere of 5% CO_2_. Subconfluent cells were subcultured every 3–4 days. H9c2 cells were seeded on 96-well plates (10,000 cells per well) and were left for 24 h to attach. Afterwards the culture medium was replaced with serum- and pyruvate-free DMEM. After 24 h of serum deprivation, the cells were essentially non-proliferating, and were used for experiments. The cells were loaded with 100 µM ferric-ammonium citrate (FAC) in serum-free medium 24 h before the experiment. The cells were then washed, and to prevent potential interferences (especially with regard to various trace elements) the medium was replaced with a buffer prepared from Millipore-filtered (demineralized) water, containing 116 mM NaCl, 5.3 mM KCl, 1 mM CaCl_2_, 1.2 mM MgSO_4_, 1.13 mM NaH_2_PO_4_, 5 mM glucose and 20 mM HEPES (pH 7.4). Cells were then loaded for 30 min at 37°C with 1 µM cell-permeable acetoxymethyl ester of calcein green (Molecular Probes/KRD, Czech Republic), and washed. Cellular esterases cleave the acetoxymethyl groups to render the cell membrane-impermeable calcein green, which fluorescence was quenched by FAC. Intracellular fluorescence (λ_ex_  =  488 nm; λ_em_  =  530 nm) was then followed for 24 hours at 37°C using an Infinite 200 M plate reader (Tecan, Austria). DEX, SOB and MER were compared to the strong lipophilic Fe chelator SIH that was used as a reference agent.

### 8. Data analysis

All data are presented as the means ± SD. Data were subjected to one-way or two-way ANOVA with Dunnett’s post-test using GraphPad Prism 5.00 (GraphPad Software, California, U.S.A.) with P≤0.05 as the level of significance. All measurements were performed in more than four independent experiments. IC_50_ values (concentration of agents inducing a 50% proliferation decrease compared to untreated controls) as well as combination index values (*CI* –a quantitative measure of the degree of drug interaction) were calculated using CalcuSyn 2.0 software (Biosoft, Cambridge, U.K.).

The IC_50_ values (concentration of agents inducing 50% proliferation decrease as compared to untreated controls or the median effect dose) were calculated as follows:

Where *F_a_* is the fraction affected (proliferation inhibited) by a drug treatment; *F_u_* is the uninhibited fraction; *D_x_* is the dose of a drug; *D_m_* is the median effect dose (IC_50_) and m is the slope of the curve. The latter software was also used to obtain the combination index (*CI*) –a rigorous quantitative measure of the degree of drug interaction:
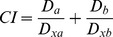



Where *D_a_* and *D_b_* are the doses of drugs used in combination, whereas *D_xa_* and *D_xb_* are the isoeffective doses. Chou and Talalay describe the drug interactions in terms of nearly additive effect (*CI* 0.9 – 1.1), slight synergism (*CI* 0.85 – 0.90), moderate synergism (*CI* 0.7 – 0.85), synergism (*CI* 0.3 – 0.7), strong synergism (*CI* 0.1 – 0.3), very strong synergism (*CI* < 0.1), slight antagonism (*CI* 1.1 – 1.2), moderate antagonism (*CI* 1.20 – 1.45), antagonism (*CI* 1.45 – 3.3), strong antagonism (*CI* 3.3 – 10) or very strong antagonism (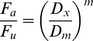 *CI* > 10). Furthermore, to assess the changes of drug–drug interactions as a function of concentration or activity, fractional affect (*F_a_*) – combination index (*CI*) plots were calculated using the CalcuSyn computer simulations.

## Results

### 1. In vitro cardiotoxicity studies

**Figure 2:**
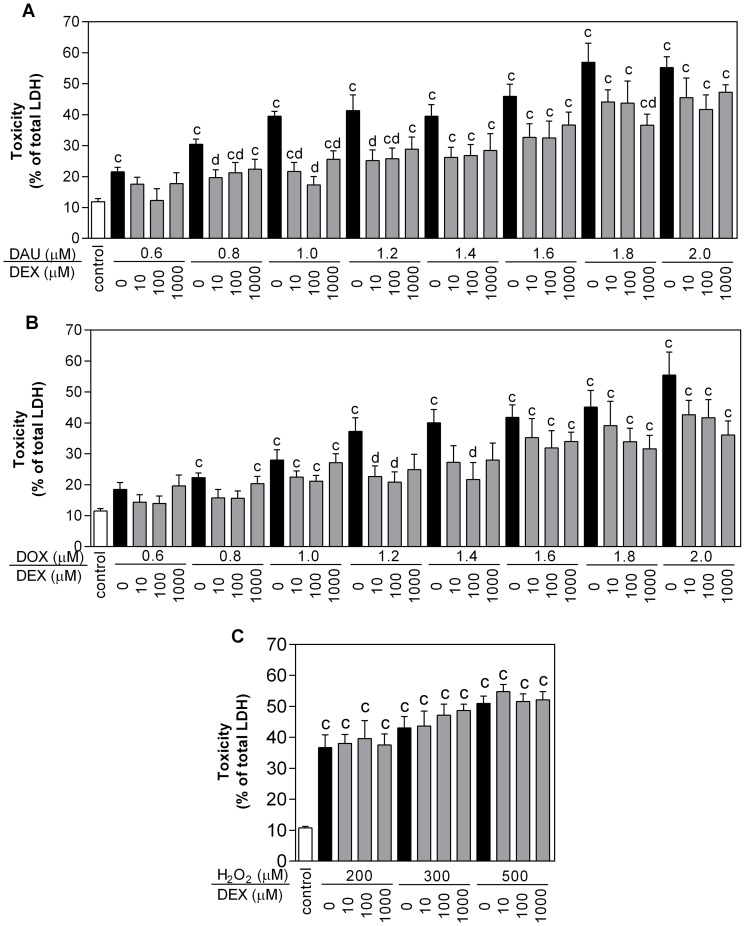
Protection of neonatal rat cardiomyocytes from toxicities induced by anthracyclines and hydrogen peroxide (H_2_O_2_). Cells were pre-incubated with three concentrations of dexrazoxane for 3 h and then co-incubated with increasing concentrations of anthracyclines daunorubicin (DAU, A) or doxorubicin (DOX, B) for 3 h following a 48-h anthracycline-free period or for 48 h with H_2_O_2_ (C). Toxicity was assessed as the % of total lactate dehydrogenase (LDH) released from cardiomyocytes into the cell culture medium. Data obtained from ≥4 independent experiments are expressed as the mean ± SD, statistical significance: c – compared with drug-free control (DMSO); d – compared with DAU or DOX; p – compared with H_2_O_2_ (one-way ANOVA with Dunnett’s post-test, P≤0.05).

**Figure 3:**
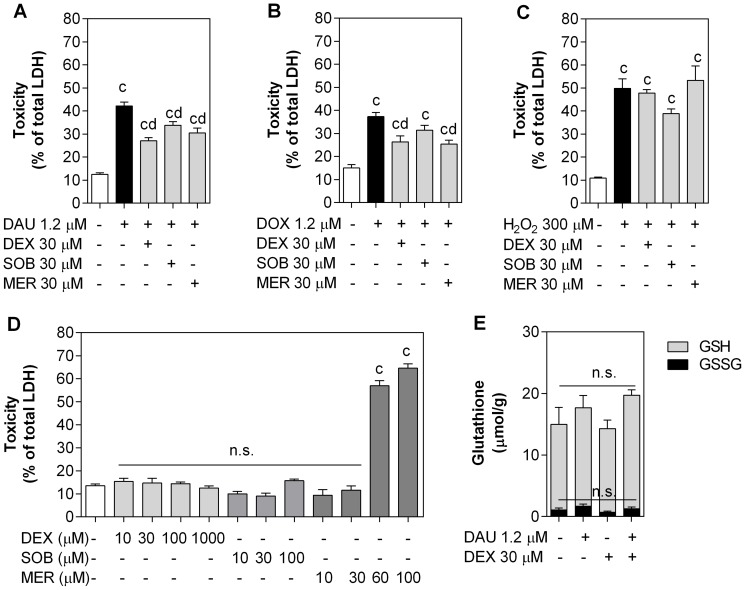
Effects of the studied substances on the viability and glutathione levels in neonatal rat cardiomyocytes. Neonatal rat cardiomyocytes were pre-incubated with dexrazoxane (DEX), sobuzoxane (SOB) or merbarone (MER) for 3 h and then incubated with anthracyclines daunorubicin (DAU, A) or doxorubicin (DOX, B) for 3 h following a 48-h anthracycline-free period or for 48 h with hydrogen peroxide (H_2_O_2_, C). DEX, SOB and MER individual toxicity after 48-h incubation is shown in panel D. Effects of DEX pre-treatment for 3 h with a 3-h DAU incubation and subsequent 48-h DAU-free period on cellular oxidised and reduced glutathione content are shown in panel E. Data obtained from ≥ 4 independent experiments are expressed as the mean ± SD, statistical significance: c – compared to control; d – compared to DAU or DOX (one-way ANOVA with Dunnett’s post-test, P≤0.05).

First, the cardiotoxic effects of ANTs and potential protective interventions were assessed using a previously published protocol [18] consisting of a 3-h exposure of NVCMs to DAU or DOX, followed by washes to remove ANTs and myocyte incubation in ANT-free medium; cellular injury was determined using an LDH release assay. As observed in Figure 2A-B, the exposure of isolated cardiomyocytes to DAU or DOX for 3 h followed by a 48-h washout period led to a statistically significant loss of viability (determined as the percentage of total LDH release) ranging from ∼20% at 0.6 µM for both ANTs to ∼55% at 2.0 µM. Pre-incubation of the cells with 10, 100 and 1000 µM DEX for 3 h induced significant protection from the toxicity of both ANTs at concentrations up to 1.2 µM (DAU) and 1.4 µM (DOX). This effect did not appear to be dose-dependent, as in most experiments the 10 µM and 100 µM DEX concentrations offered better protection than 1000 µM (Figure 2A-B). In cardiomyocytes exposed to the oxidative stress-inducing agent H_2_O_2_, toxicity ranging from ∼36% at 200 µM to ∼50% at 500 µM H_2_O_2_ was detected. However, no significant protection against H_2_O_2_-induced toxicity was found with any assayed concentration of DEX (Figure 2C). For further examinations, 1.2 µM DAU or DOX were chosen, as in this concentration both ANTs caused significant toxicity preventable by DEX preincubation. Accordingly, 300 µM H_2_O_2_ was chosen for comparison, as this concentration caused comparable toxicity to 1.2 µM of DAU or DOX. Of the three TOP2 catalytic inhibitors used in this study, DEX (10 – 1000 µM) and SOB (in concentrations up to its solubility limit of 300 µM) did not cause any significant loss of NVCM viability. Conversely, MER exhibited significant toxicity at concentrations ≥60 µM (Figure 3D). DEX, SOB and MER were then compared for their cardioprotective potential at a concentration of 30 µM, i.e., the highest concentration at which none of the drugs induced its own toxicity. All three TOP2 catalytic inhibitors were able to partially but significantly protect NVCMs against 1.2 µM DAU or DOX with ∼10 – 15% toxicity reduction in comparison with DAU or DOX toxicity alone, but the TOP2 inhibitors had no significant effect on the toxicity caused by 300 µM H_2_O_2_ (Figure 3A-C).

As observed in Figure 3E, the incubation of NVCMs with 1.2 µM DAU for 3 h followed by 48 h in DAU-free medium did not lead to a statistically significant increase in either GSH or GSSG, despite the significant toxicity caused by this concentration and incubation schedule. The pre-incubation of cardiomyocytes with 30 µM DEX for 3 h followed by co-incubation with DAU showed an insignificant trending increase in GSH, but this occurred in the absence of changes to GSSG. The control incubation with DEX alone did not induce any significant difference from control values for either GSH or GSSG (Figure 3E).

A three-hour exposure of NVCMs to 1.2 µM DAU or DOX caused marked changes in cardiomyocyte morphology, including cytoplasmic vacuolisation and granulation, which proceeded into discontinuation of the cellular monolayer and cellular and nuclear shrinkage. Cardiomyocytes were stained with the JC-1 probe, and its signal was visualised by fluorescence microscopy. Following exposure to DAU or DOX, there was a conspicuous transition of normal red-stained mitochondria with polarised inner membranes to diffuse green fluorescence indicating dying cells with depolarised mitochondria. Pre-incubation with 30 µM DEX, SOB and, to a lesser extent, MER, partially prevented both ANT-induced changes in cellular morphology as well as the dissipation of the mitochondrial inner membrane potential (Figure S1).

### 2. Proliferation studies

**Figure 4:**
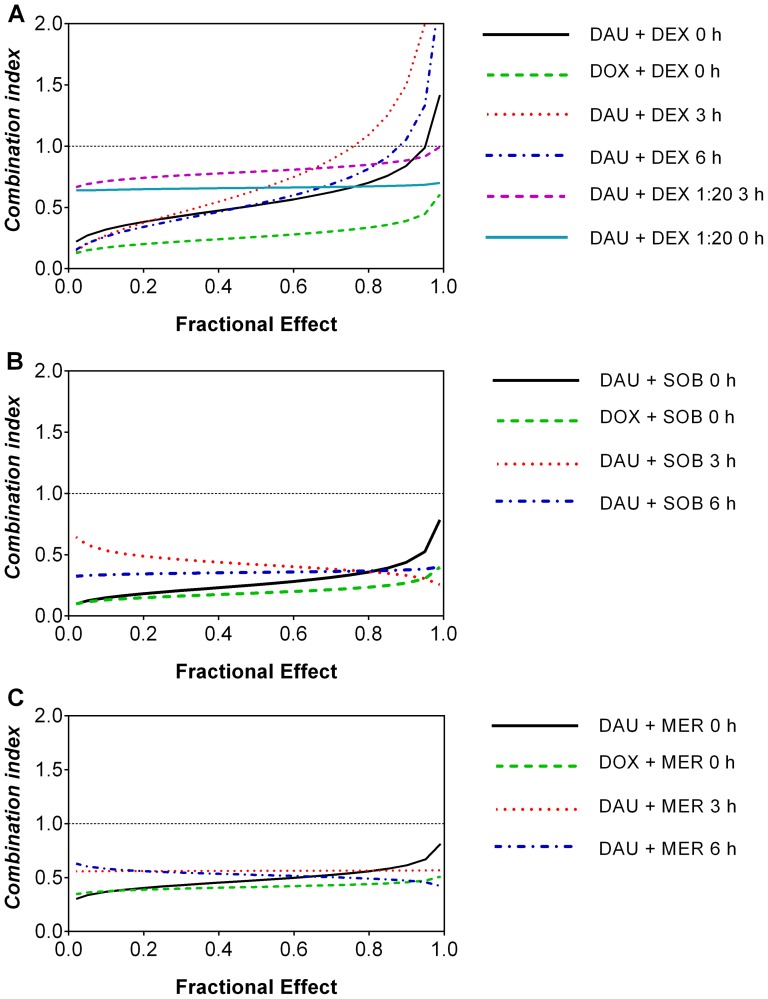
Effects of dexrazoxane, sobuzoxane or merbarone and daunorubicin or doxorubicin on the proliferation of HL-60 cell line. Cells were incubated either continuously with dexrazoxane (DEX, A), sobuzoxane (SOB, B) or merbarone (MER, C) and daunorubicin (DAU) or doxorubicin (DOX) for 72 h or pre-incubated with DEX (A), SOB (B) or MER (C) for 3 h or 6 h and then incubated for 72 h with all drugs at concentrations corresponding to their IC_50_ values and IC_50_ fractions and multiples (1/8; 1/4; 1/2; 1; 2; 4). Alternatively, cells were either co-incubated for 72 h or pre-incubated with DEX for 3 h and then co-incubated with DAU for 72 h at a ratio of 1:20 DAU:DEX (A). Computer simulations of combination index (*CI*) - cellular proliferation (fraction affected - *Fa*) dependences were obtained using CalcuSyn 2.0 software.

After incubation for 72-h, all substances examined had significant and dose-dependent antiproliferative effects on HL-60 cells. The ANTs (DAU and DOX) were effective at concentrations that were three orders of magnitude lower than the catalytic inhibitors; the IC_50_ values for all drugs were as follows: 19 nM for DAU, 38 nM for DOX, 25 µM for DEX, 48 µM for SOB and 38 µM for MER. Single agents and combinations of ANTs and TOP2 catalytic inhibitors were then incubated for 72 h at concentrations corresponding to their IC_50_ values, and their interactions were analysed according to the Chou-Talalay method. Additionally, as drug-drug interactions can change as a function of concentration or activity, we also examined combinations of chelators and anticancer drugs at fractions and multiples (1/8; 1/4; 1/2; 1; 2; 4) of their IC_50_ values, and Fraction affected (*Fa*) – combination index (*CI*) plots were calculated using computer simulations (Figure 4). Proliferation data are shown in panels A and B in Figures S2–S4, and combination index (*CI*) calculated values are summarised in Tables S1–S3. These analyses revealed that DEX, SOB and MER potentiated the antiproliferative effects of both DAU and DOX when these substances were co-incubated simultaneously, as the corresponding *CI* values ranged from moderate to strong synergism.

In another series of experiments, DEX, SOB and MER were pre-incubated for 3 or 6 h prior to the addition of DAU with a subsequent 72-h co-treatment (panels C and D in Figures S2–S4, Tables S1-S3). As observed in these figures and tables, these drug combinations remained synergistic with the exception of combinations of DAU with DEX at its highest concentration; in this case, the *CI* values were additive or in one rare case moderately antagonistic. Additionally, apart from the standard Chou-Talalay combination study design (where the agents are applied at equipotent doses), DAU and DEX were also examined using the fixed 1:20 concentration ratio used in clinical practice [19]. While rather less pronounced synergism was detected for lower concentrations, here, no antagonism occurred, even following preincubation with DEX for 3 h, and in this setting, all concentrations of DEX potentiated the antiproliferative activity of DAU towards HL-60 cells (Figure S2E-F, Table S1).

### 3. Caspase activity assays

**Figure 5:**
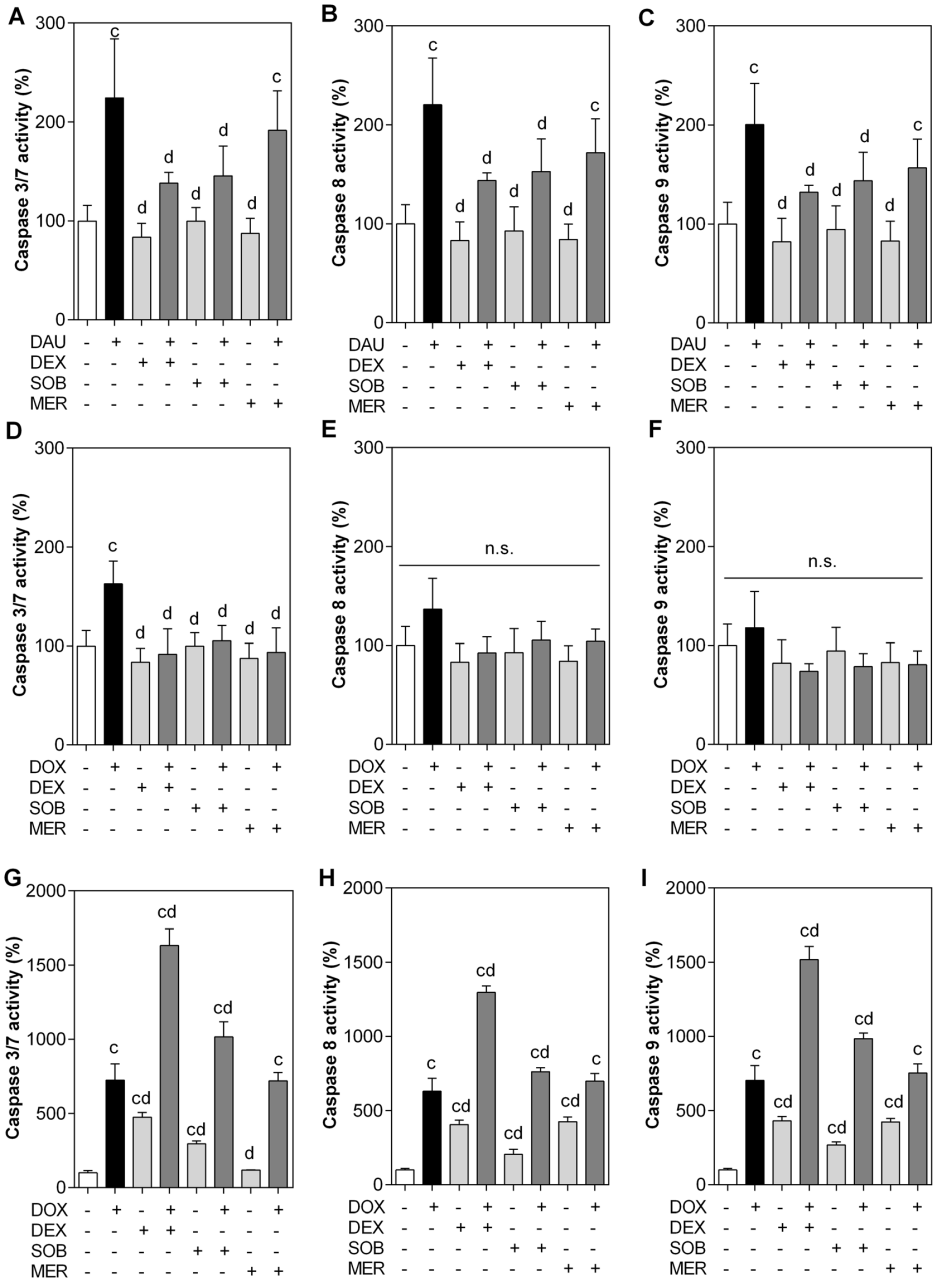
Caspase activities in cardiomyocytes and the HL-60 cell line. Neonatal rat cardiomyocytes (A - F) were pre-incubated with 30 µM dexrazoxane (DEX), sobuzoxane (SOB) and merbarone (MER) for 3 h and then co-incubated with 1.2 µM daunorubicin (DAU; A - C) or doxorubicin (DOX; D - F) for 3 h, followed by an anthracycline-free 48-h period. The HL-60 cell line (G - I) was co-incubated with 25 µM DEX, 38 µM SOB, 48 µM MER and 19 nM doxorubicin (DOX) for 72 h. The activity of caspases 3/7 (A, D, G), 8 (B, E, H) and 9 (C, F, I) was assessed with a chemiluminescence assay as described in the materials and methods, corrected for protein content and expressed as the percentage of untreated control cells (100%). Data from ≥4 experiments are expressed as the mean ± SD, statistical significance c – compared to control; d – compared to DAU or DOX; n.s. – not significant (one-way ANOVA with Dunnett’s post-test, P≤0.05).

Combinations of ANTs and TOP2 catalytic inhibitors were also examined for their ability to activate caspases, which are key mediators of apoptosis. Incubation of NVCMs with 1.2 µM DAU for 3 hours followed by 48 h of DAU-free period caused significant activation of caspases 8 and 9, which are the key extrinsic (receptor-mediated) and intrinsic (mitochondrial) apoptotic pathway initiators, respectively, as well as the execution caspases 3/7 to ∼200% of control values. Incubation with 30 µM concentrations of all three TOP2 catalytic inhibitors did not cause any significant increase in the activity of any caspase, but DEX and SOB significantly protected cardiomyocytes against caspase activation by DAU. Although MER pre-treatment also showed a trend towards decreased activation of all caspases, this did not reach statistical significance compared to the DAU group. Consistent with the results of the LDH leakage assay, the activation of caspases after incubation with DOX was generally less pronounced than after DAU incubation. The activation of both initiation caspases was insignificant compared with the control (Figure 5E-F), although there was a slight increase. Overall, preincubation of the cells with DEX, SOB or MER did not significantly change initiation caspase activation, despite the trend towards lower activity. However, the activity of the executive caspase 3/7 was significantly increased following DOX treatment, while TOP2 catalytic inhibitors effectively prevented this change (Figure 5D).

In HL-60 cells, the activity of all caspases (3/7, 8 and 9) was significantly increased by all the compounds (with the exception of MER and caspase 3/7) after 72-h incubations at concentrations corresponding to their IC_50_ values. For individual caspases, these increases ranged from ∼200% to ∼500% for DEX, SOB and MER and ∼600% to 700% for DOX. The combinations of DOX with DEX or SOB increased caspase activity significantly in comparison to both control and individual drugs alone. DOX combined with MER did not induce a significant increase in caspase activation compared to DOX alone, nor was the activation caused by DOX alone mitigated by MER co-treatment (Figure 5I).

### 4. Effects of treatments on the cell cycle in HL-60 cells

**Figure 6:**
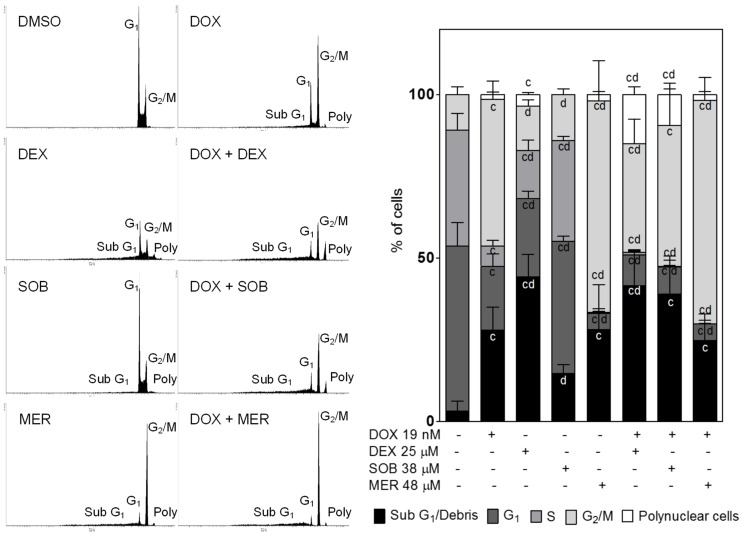
Cell-cycle analyses. The human acute promyelocytic leukaemia HL-60 cell line was incubated with dexrazoxane (DEX), sobuzoxane (SOB), merbarone (MER) and doxorubicin (DOX) and combinations thereof in concentrations corresponding to their IC_50_ values for 72 h and then subjected to flow cytometry cell cycle analysis as described in the materials and methods. Data from 4 independent experiments are expressed as the mean ± SD, statistical significance: c – compared to untreated control cells; d – compared to DOX-treated cells (one-way ANOVA with Dunnett’s post-test, P≤0.05).

Control HL-60 cell populations in our experiments consisted of ∼50% G_1_ cells, ∼5% cells in S phase, and ∼10% cells in G_2_/M phase of the cell cycle. The remaining ∼5% constituted dead cells (sub-G_1_ population). A 72-h incubation with 19 nM DOX (a concentration corresponding to its IC_50_ value) caused significant reductions in the G_1_ and S phase populations (approximately a 1/3 drop for each) in favour of the G_2_/M phase (∼1/3 increase), sub-G1 population (∼1/4 increase) and polynuclear cells (∼2%). Incubation with the IC_50_ DEX concentration (25 µM) also induced a drop in the G_1_ and S phase cell populations; this drop was, however, significantly lower than that following DOX treatment (26% vs. 20%, respectively). The number of cells in G_2_/M arrest was also significantly lower than in the DOX group. The sub-G_1_ population (∼45%) for the DEX group was significantly higher than both the control and DOX-treated cells, and the polynuclear population was also significantly higher (∼5%) than in control cells. In contrast, no polynuclear cells were found following the incubation of HL-60 cells with 38 µM SOB. Additionally, despite a slight trend, there was no significant G_2_/M phase arrest. Significant decreases in the G_1_ and S phase cell populations occurred in comparison to the control; however, these populations were simultaneously significantly higher than for the DOX-treated group. MER (48 µM) treatment induced the most pronounced G_2_/M arrest of all the drugs tested in monotherapy (∼65%), and this result was significantly higher than both the control and DOX treatment. MER also caused the most pronounced decrease in the G_1_ and S phase populations (∼45% and ∼35% decrease compared to the control, respectively). The sub-G_1_ population increased over the control values to a similar extent as DOX treatment (28%). The induction of polyploidy (2% of polynuclear cells) was also similar to DOX. The combination of DOX with DEX or SOB increased the sub-G_1_ population but not significantly more than DOX alone. Both DEX and SOB in combination with DOX caused a marked decrease in G_1_ (∼10% each) and S (1 and 0.2%, respectively) phase cells together with a marked increase in the polynuclear cell population (15 and 10%, respectively). The combination of DOX with MER did not induce any significant changes compared to MER treatment alone (Figure 6).

### 5. Intracellular Fe chelation assessments

**Figure 7:**
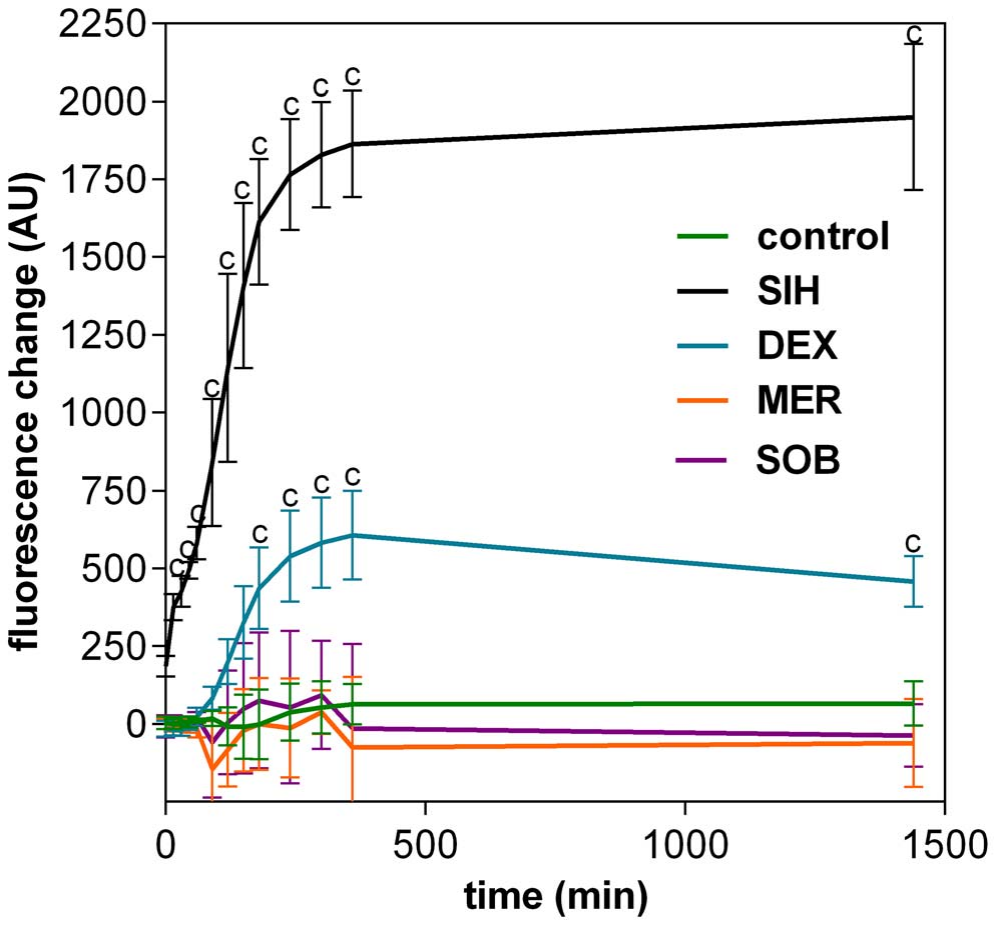
Intracellular iron chelation properties. The intracellular iron (Fe) chelation by dexrazoxane (DEX), sobuzoxane (SOB) and merbarone (MER) was measured as the rate of Fe displacement from the Fe-calcein complex in H9c2 cardiomyoblast cell line. The change of fluorescence of the intracellular-trapped calcein in the H9c2 cells loaded with 100 µM ferric-ammonium citrate was assessed after adding the studied substances (100 µM) as described in Materials and methods section. The experimental strong Fe chelator SIH was used as a positive control. Data from 4 independent experiments are expressed as mean ± SD. Statistical significance: Two-way ANOVA Dunnet’s post-hoc test, P≤0.05; c–compared to control.

The abilities of DEX, SOB and MER to chelate labile intracellular Fe ions were assessed fluorimetrically using the calcein-AM assay [20]. In H9c2 cells, a typical result was obtained with experimental Fe chelator SIH [21], which increased the intracellularly-trapped calcein fluorescence significantly compared to control from the start of incubation. DEX also increased significantly the calcein fluorescence compared to control, but only to ∼30% of the levels achieved with SIH. Moreover, a substantial lag-time was noticed as significant change in fluorescence compared to control was established only after ∼2.5 hours. Neither SOB nor MER were able to significantly displace Fe from its calcein-complex compared to control (Figure 7).

## Discussion

During the last decade, both the traditional "ROS and Fe" hypothesis of ANT-induced cardiotoxicity as well as the notion that DEX protects cardiomyocytes *via* its Fe-chelating metabolite ADR-925 have been subjects of renewed discussion and growing controversy. Classical antioxidants and ROS scavengers (such as N-acetylcysteine, coenzyme Q10, vitamins E and C), although usually very effective in acute ANT cardiotoxicity experiments, have failed in all controlled and randomised clinical trials conducted so far, although there were often notable discrepancies in the concentrations/doses of antioxidants used in *in vitro* and *in vivo* experiments as well as differences in the responses within various animal species [22], [23], [24]. Furthermore, several Fe chelators that are both stronger and more selective for Fe than ADR-925 have failed to match the unique and experimental model-independent cardioprotective efficacy of DEX [23]. Of significance, ADR-925 complexed with Fe may promote redox reactions rather than inhibiting them [25], and ADR-925 failed to protect isolated cardiomyocytes [18]. In a model of chronic DAU-induced heart failure in rabbits, DEX was able to prevent increased cardiomyocyte apoptosis in hearts, but this protection was not accompanied by a significant reduction in lipoperoxidation [26], suggesting that oxidative stress may merely be a secondary by-product that accompanies ANT cardiotoxicity and subsequent heart failure rather than a primary culprit. Furthermore, a close derivative of DEX (ICRF-161) that lacks TOP2 inhibitory action but can be activated to produce a diacid diamide metabolite with ability to displace Fe from the complex with DOX (although with less efficiency than ADR-925) has been found to be ineffective against chronic ANT cardiotoxicity in rats when compared to DEX [27]. Thus, there has been a search for alternative or additional hypotheses to explain the mechanisms of both ANT cardiotoxicity as well as DEX-induced cardioprotection.

The effective cardioprotective properties of DEX have been demonstrated by many clinical studies [19] and were firmly confirmed with a recent meta-analysis [22]. However, focused *in vitro* analyses of DEX cardioprotection and the mechanism(s) involved have been scarce. Hasinoff *et al*. [28] found that pre-incubation with DEX (90 µM, 3 h) protected cardiomyocytes from mitochondrial membrane potential loss induced by relatively low concentrations of DOX (0.15 – 0.5 µM, 3 h incubation followed by 48 h post-incubation in DOX-free media). In the same study, the authors showed that signs of apoptosis are increased by approximately 200% after 48 h treatment with clinically relevant concentrations of DOX (0.2 and 1 µM), which is comparable to our results obtained under similar conditions. In the present study, we employed a wide range of concentrations of DEX (10–1000 µM) as well as two different ANTs (DAU and DOX, 0.6–2.0 µM) to describe in detail the effects of these drugs at concentrations that can be present in the plasma of patients after drug administration [29]. Furthermore, we directly compared these results in a model of oxidative insult induced by H_2_O_2_ (200–500 µM), which resulted in viability loss comparable to both ANTs. Although DEX was able to consistently reduce LDH release from cardiomyocytes induced by DAU or DOX, it demonstrated no protection against any concentration of H_2_O_2_ employed in this study. Furthermore, the protective effects of DEX against DAU-induced toxicity were not associated with changes to GSH or GSSG within cardiomyocytes, which suggests that these effects are oxidative stress-independent. These results agree well with Lyu *et al*. [30], who demonstrated that 200 µM DEX protected the H9c2 cardiomyoblast-derived cell line from DOX (0.5–5 µM), but not H_2_O_2_- or camptothecin-induced DNA double strand breaks as determined by elevated γ-H2AX expression. At the same time, vitamin C and N-acetylcysteine did not protect these cells against DOX. These data are also in good agreement with our previous study showing that the glutathione antioxidant system was not affected by DAU treatment either *in vitro* in H9c2 cells or *in vivo* using a chronic ANT cardiotoxicity model [31]. Additionally, in the present study, we observed no effects of DAU, DEX or their combination on oxidised or reduced glutathione cellular levels in NVCMs.

The use of DEX in clinical practice has been relatively limited. One of the major reasons for this is attributed to doubts concerning the possible interference of this drug with ANT anticancer effects. This question becomes even more urgent considering the possibility that ANTs and DEX may share TOP2 as a molecular target both for their anticancer and potentially cardioprotective actions. To examine this issue, we used a model with the HL-60 human promyelocytic leukaemia cell line and performed thorough analyses of combinations of DEX with DAU or DOX following 72-h co-incubations using the rigorous Chou-Talalay method [13]. The obtained *CI* values demonstrated the synergistic antiproliferative effects of DEX with DAU or DOX, mainly at lower concentrations. Previously, using Chinese hamster ovary cells (which are neither cancerous nor cardiac), one study reported that DEX might exhibit antagonism of DOX- or DAU-mediated growth inhibition when cells were pre-incubated with DEX before ANT was added [32]. Therefore, an additional series of experiments was undertaken under conditions similar to those in previous cardioprotective experiments. The pre-incubation of HL-60 cells for 3 h with DEX prior to DAU caused a trend towards a less synergistic antiproliferative interaction. At most concentrations, the *CI* values were still indicative of synergistic effects; however, combinations at the highest concentrations (2x and 4x IC_50_) resulted in nearly additive effects or moderate antagonism. Surprisingly, prolonged pre-incubation with DEX for 6 h did not result in further aggravation of the combination outcomes; rather, in comparison to the 3-h pre-incubation period, the *CI* values were consistently lower. The standard Chou and Talalay combination study design involves the application of examined agents at equipotent doses (IC_50_ concentrations and their multiples and fractions). However, as the IC_50_ values of the ANTs and DEX differ by three orders of magnitude, we also assessed their combination at the ratio of 1:20 ANT to DEX used in clinical practice [19]. At this fixed concentration ratio, we obtained synergistic outcomes as well. Interestingly, even a 3-h DEX pre-incubation prior to DAU did not result in antagonism with this ratio, although the *CI* values were consistently higher than for combination experiments with no pre-incubation.

Apoptosis is the most important form of programmed cell death, and it is considered to be an important component of both ANT tumoricidal action as well as cardiotoxicity [2]. In this study, we observed significant caspase activation in both cardiomyocytes as well as in HL-60 cells. In both cell types, there were non-specific increases in both the executive downstream caspases 3 and 7 and the initiation caspases 8 and 9, suggesting a concomitant involvement of both intrinsic (mitochondrial) as well as extrinsic (receptor-mediated) apoptotic pathways and/or crosstalk between them. Pre-incubation with DEX reduced ANT-induced caspase activation in cardiomyocytes, whereas the marked activation of all assayed caspases induced by 72-h DOX treatment was not blunted but rather further enhanced by DEX in HL-60 cells. This striking difference in DEX-induced apoptotic signalling in heart and cancer cells may be of considerable importance, and its molecular mechanisms deserve further study.

The incubation of HL-60 cells with DEX caused significant depletion of G_1_ and S-phase cells. Conversely, the sub-G_1_ cell population, which is composed of cell debris, as well as the polynuclear cell population were significantly increased. When cells were co-incubated with DOX, the G_1_ and S-phase cell depletion was even more pronounced, and the proportion of polynuclear cells and cell debris was significantly higher than with DOX treatment alone. Therefore, these results strongly support the notion that DEX does not reduce the antineoplastic ability of ANTs, which is in line with previous clinical data obtained in a leukaemia setting as well as the conclusions of a recent meta-analysis of all randomised clinical trials performed thus far [22].

The group of TOP2 catalytic inhibitors consists of many compounds with various chemical structures, including bisdioxopiperazines (DEX, SOB), fostriecin, aclarubicin, suramin, novobiocin, and MER, as well as numerous other experimental agents [33]. Unlike TOP2 poisons, catalytic inhibitors do not promote double-strand break formation and thus do not cause permanent DNA damage [33]. SOB (MST-16) is a pro-drug of ICRF-154, a bisdioxopiperazine DEX analogue. It has been licensed in Japan for the treatment of blood neoplasms, in particular malignant lymphoma and adult T-cell leukaemia [34]. According to unpublished data quoted by Inutsuka *et al.* [35], SOB has been shown to be protective against DOX cardiac and renal toxicity in rats. Additionally, SOB (750 mg/kg) exhibited synergy with DOX (7.5 mg/kg) in colon-26 tumour-bearing mice as measured by prolonged survival of the animals. In the same study, the authors observed an increased G_2_/M population (by 10%) after incubating colon-26 cells with a combination of SOB (10 µM) and DOX (0.1 µM) for 24 h compared to DOX-treated samples [35]. The G_2_/M arrest was also reported in a human mammary tumour cell line (MDA-MB-435) exposed to 4 µM SOB for 20 h [36]. Given the gradual increase in ANT sensitivity as cells transition from relatively resistant G_1_ phase to G_2_/M phase, which was observed in exponentially growing Molt-4 cells [37], the accumulation of G_2_/M cells before ANT treatment could be beneficial as the relatively resistant population is depleted in favour of the more sensitive population. In our study, SOB alone did not induce significant G_2_/M phase arrest of HL-60 cells after treatment for 72 h. However, SOB significantly reduced the G_1_ and S phase populations, which could be favourable for increasing HL-60 cell sensitivity to DOX. The combination of SOB with DOX caused even more pronounced depletion of G_1_ and S phase cells together with an increase in polyploid cells and cell debris. Indeed, the combination of DOX or DAU with SOB in our study provoked unequivocally synergistic antiproliferative effects in HL-60 cells at all assessed concentrations and schedules (including 3- and 6-h SOB pre-incubations), and SOB also enhanced the activation of caspases induced by DOX. Conversely, SOB pre-incubation with NVCMs led to significant protection from DAU- and DOX-induced toxicity as assessed by LDH release, mitochondrial membrane potential measurements and caspase activity measurements.

MER is a non-sedative barbiturate derivative that is structurally unrelated to bisdioxopiperazines [38]. Its principal effect on replicating cells is the inhibition of chromosome condensation, activation of c-Jun and JNKs and induction of G_2_/M cell cycle blockade [33]. In this study, although MER significantly protected NVCMs against both DAU- and DOX-induced LDH release, it also induced some toxicity in NVCMs. MER insignificantly reduced the DAU-induced activation of caspases and also only partially protected cardiomyocytes from ΔΨ_m_ loss. However, of the three catalytic inhibitors examined in this study, MER showed the highest potential to act synergistically with ANTs in HL-60 cells, particularly at higher concentrations for which there was no tendency towards increased *CI* values. Additionally, with 3- or 6-h pre-incubations, the drug combinations remained consistently synergistic. The effects of MER on the cell cycle in HL-60 cells differed considerably from the bisdioxopiperazines. MER alone caused dramatic G_2_/M arrest, which was even more pronounced than that induced by DOX. In addition, the sub G_1_ and polynuclear populations increased. However, unlike DEX or SOB, the MER + DOX combination did not induce more pronounced cell cycle changes.

As DEX and SOB are the members of the bis-dioxopiperazine family, they can be metabolized to Fe-chelating compounds. Whereas DEX is well known to undergo metabolization to ADR-925, SOB must be probably first metabolized to ICRF-154 and then to the open-ring product, thus requiring two-step metabolism to metal chelator. To examine this issue, the Fe intracellular chelating properties of DEX, SOB and also MER were examined in H9c2 rat embryonic cardiomyoblast-derived cell line using the measurements of calcein-AM fluorescence intensity to compare the cardioprotection with their ability to chelate Fe. The experimental strong lipophilic Fe chelator SIH was repeatedly documented to chelate free Fe both in solution as well as in cells, and to displace Fe from its complex with calcein resulting in dequenching of its fluorescence [21], [15] and therefore it has been used as the reference chelator in our study. Indeed, fast increase in calcein fluorescence could be observed upon addition of 100 µM SIH to the cells with intracellular-trapped calcein-Fe complex and also DEX is able to significantly chelate Fe in comparison with control, although rather slowly and with only ∼30% efficiency as compared to SIH. The ∼2.5 h lag-time before the start of fluorescence increase was probably caused by the need of DEX hydrolysis to the chelating metabolite ADR-925. Similar result was observed by Hasinoff et al. [18], who found that unlike ADR-925, which displaces Fe from the cell-trapped calcein-Fe complex in a pattern similar to SIH, DEX chelated Fe only partially and with substantial lag-time. In our study, neither SOB nor MER had any ability to displace Fe from the calcein complex as significant increase of fluorescence was not observed in the comparison with control. Hence, these data are in agreement with previous suggestions that Fe chelation may not be an important factor of cardioprotective efficiency [23]. It should be noted, however, that it is still possible that Fe chelation and/or antioxidant action takes place in some particular cellular compartments and/or time periods that is not readily determined by currently available assays and this deserves further examinations.

Mammalian cells contain two distinct TOP2 isoforms. The alpha isoform (TOP2A) is present in high amounts in proliferating and undifferentiated cells, and its expression is cell cycle-dependent, reaching a maximum in G_2_/M phase. Conversely, the beta isoform (TOP2B) is expressed at relatively steady levels throughout the cell cycle and is the major TOP2 form in quiescent cells. Whereas TOP2A is implicated predominantly in DNA replication, TOP2B has important roles in the regulation of gene transcription [39], [40]. These marked differences in the expression, function and regulation of the TOP2 isoforms in cardiac and cancer cells may be responsible for differential modulation of ANT toxicity. Lyu *et al.* [30] detected preferential proteasomal degradation of TOP2B isoform in H9c2 cardiomyoblast-derived cells after DEX exposure, coupled with a lack of significant changes to TOP2A. The selective degradation of TOP2B could prevent ANT-induced damage to the cardiomyocyte genome due to poisoning by this isoform and subsequent DNA double-strand breaks while sparing the TOP2A isoform in cancer cells. Additionally, a truncated form of TOP2B was discovered in mitochondria [41], providing a plausible explanation for ANT-induced damage (as well as DEX-mediated protection) of cardiac mitochondria, organelles that are known to be important targets of ANT-induced cardiotoxicity [23]. The most straightforward argument for the TOP2B isoform as a mediator of DEX-induced cardioprotection is the recent study from Zhang *et al.* [10] reporting that the cardiomyocyte-specific deletion of the *Top2b* gene protected mice from developing DOX cardiotoxicity. Interestingly, preferential inhibition of the TOP2A isoform has been suggested for MER [33], which may correspond to the relatively weaker cardioprotective efficacy seen with this drug in this study compared to DEX or SOB.

In conclusion, our results demonstrate that the TOP2 catalytic inhibitors DEX, SOB and MER differentially and favourably modulate toxicity of DAU or DOX to cardiac and leukemic cancer cells. One plausible explanation for these effects could involve the targeting of different TOP2 isoforms (alpha and beta) in these cells. Catalytic TOP2 inhibitors may directly protect the heart from ANT cardiotoxicity but also potentially sensitise cancer cells to ANTs, which in turn may permit ANT dose reduction with secondary reduction of cardiotoxicity risk. Taken together, this study provides a strong rationale for further investigations of TOP2 catalytic inhibitors as cardioprotectants against ANT cardiotoxicity.

## Supporting Information

Figure S1 **Effects of studied substances on cardiomyocyte morphology and mitochondrial depolarisation.** Neonatal ventricular cardiomyocytes were pre-treated with 30 µM dexrazoxane (DEX), sobuzoxane (SOB) or merbarone (MER) for 3 h, then incubated with 1.2 µM of either daunorubicin (DAU) or doxorubicin (DOX) for 3 h, followed by an anthracycline-free incubation period for 48 h. Upper panels – brightfield phase contrast photomicrographs, lower panels – darkfield epifluorescence images of the same cells taken after loading with the JC-1 probe (red emission reflects mitochondrial inner membrane potential-dependent accumulation of probe dimers in actively respiring mitochondria, green fluorescence indicates monomers of the probe released into the cytoplasm after mitochondrial depolarisation, lack of fluorescence reflects probe release from necrotic or late-stage apoptotic cells). Scale bars represent 100 µm; all panels taken at the same magnification.
(TIF)

Figure S2 **Effects of dexrazoxane and daunorubicin or doxorubicin on the proliferation of HL-60 cell line.** Cells were incubated either continuously with dexrazoxane (DEX) and daunorubicin (DAU) or doxorubicin (DOX) for 72 h (A – B) or pre-incubated with DEX for 3 h (C) or 6 h (D) and then incubated for 72 h with all drugs at concentrations corresponding to their IC_50_ values and IC_50_ fractions and multiples (1/8; 1/4; 1/2; 1; 2; 4). Alternatively, cells were either co-incubated for 72 h (E) or pre-incubated with DEX for 3 h (F) and then co-incubated with DAU for 72 h at a ratio of 1:20 DAU:DEX. Data from 4 independent experiments expressed as the mean ± SD, statistical significance: c – compared to control; d – compared to DAU or DOX (one-way ANOVA with Dunnett’s post-test, P≤0.05).
(TIF)

Figure S3 **Effects of sobuzoxane and daunorubicin or doxorubicin on the proliferation of HL-60 cell line.** Cells were incubated either continuously with sobuzoxane (SOB) and daunorubicin (DAU, A) or doxorubicin (DOX, B) for 72 h or pre-incubated with SOB for 3 h (C) or 6 h (D) and then incubated for 72 h with SOB and DAU at concentrations corresponding to their IC_50_ values and IC_50_ fractions and multiples (1/8; 1/4; 1/2; 1; 2; 4). Data from 4 independent experiments expressed as the mean ± SD, statistical significance: c – compared to control; d – compared to DAU or DOX (one-way ANOVA with Dunnett’s post-test, P≤0.05).
(TIF)

Figure S4 **Effects of merbarone and daunorubicin or doxorubicin on the proliferation of HL-60 cell line.** Cells were incubated either continuously with merbarone (MER) and daunorubicin (DAU, A) or doxorubicin (DOX, B) for 72 h or pre-incubated with MER for 3 h (C) or 6 h (D) and then incubated for 72 h with MER and DAU at concentrations corresponding to their IC_50_ values and IC_50_ fractions and multiples (1/8; 1/4; 1/2; 1; 2; 4). Data from 4 independent experiments expressed as the mean ± SD, statistical significance: c – compared to control; d – compared to DAU or DOX (one-way ANOVA with Dunnett’s post-test, P≤0.05).
(TIF)

Table S1 **The quantitative assessments of antiproliferative activities of combinations of doxorubicin (DOX) or daunorubicin (DAU) with dexrazoxane (DEX).** The HL-60 cells were incubated with DEX without pre-incubation (DEX 0 h), or with 3-hour (DEX 3 h) or 6-hour pre-incubation (DEX 6 h) and then incubated with doxorubicin (DOX) or daunorubicin (DAU) in concentrations corresponding to their IC_50_ values and IC_50_ fractions and multiples (1/8; 1/4; 1/2; 1; 2; 4) or in a fixed 1:20 DAU:DEX concentration ratio. Values of combination indexes (CI) were calculated according to the method of Chou and Talalay as described in materials and methods using Calcusyn for Windows 2.0. CI < 1, ≈ 1 or > 1 means synergism, additive effect or antagonism, respectively. Data from four experiments are expressed as mean ± SD.
(DOC)

Table S2 **The quantitative assessments of antiproliferative activities of combinations of doxorubicin (DOX) or daunorubicin (DAU) with sobuzoxane (SOB).** The HL-60 cells were incubated with SOB without pre-incubation (SOB 0 h), or with 3-hour (SOB 3 h) or 6-hour pre-incubation (SOB 6 h) and then incubated with doxorubicin (DOX) or daunorubicin (DAU) in concentrations corresponding to their IC_50_ values and IC_50_ fractions and multiples (1/8; 1/4; 1/2; 1; 2; 4). Values of combination indexes (*CI*) were calculated according to the method of Chou and Talalay as described in materials and methods using Calcusyn for Windows 2.0. *CI* < 1, ≈ 1 or > 1 means synergism, additive effect or antagonism, respectively. Data from four experiments are expressed as mean ± SD.
(DOC)

Table S3 **The quantitative assessments of antiproliferative activities of combinations of doxorubicin (DOX) or daunorubicin (DAU) with merbarone (MER).** The HL-60 cells were incubated with MER without pre-incubation (MER 0 h), or with 3-hour (MER 3 h) or 6-hour pre-incubation (MER 6 h) and then incubated with doxorubicin (DOX) or daunorubicin (DAU) in concentrations corresponding to their IC_50_ values and IC_50_ fractions and multiples (1/8; 1/4; 1/2; 1; 2; 4). Values of combination indexes (*CI*) were calculated according to the method of Chou and Talalay as described in materials and methods using Calcusyn for Windows 2.0. *CI* < 1, ≈ 1 or > 1 means synergism, additive effect or antagonism, respectively. Data from four experiments are expressed as mean ± SD.
(DOC)
