# Supplementary figures and images for: Catalytic Inhibitors of Topoisomerase II Differently Modulate the Toxicity of Anthracyclines in Cardiac and Cancer Cells
Source: PLoS One. 2013 Oct 7;8(10):e76676. doi: 10.1371/journal.pone.0076676 (PMC3792022; doi:10.1371/journal.pone.0076676)

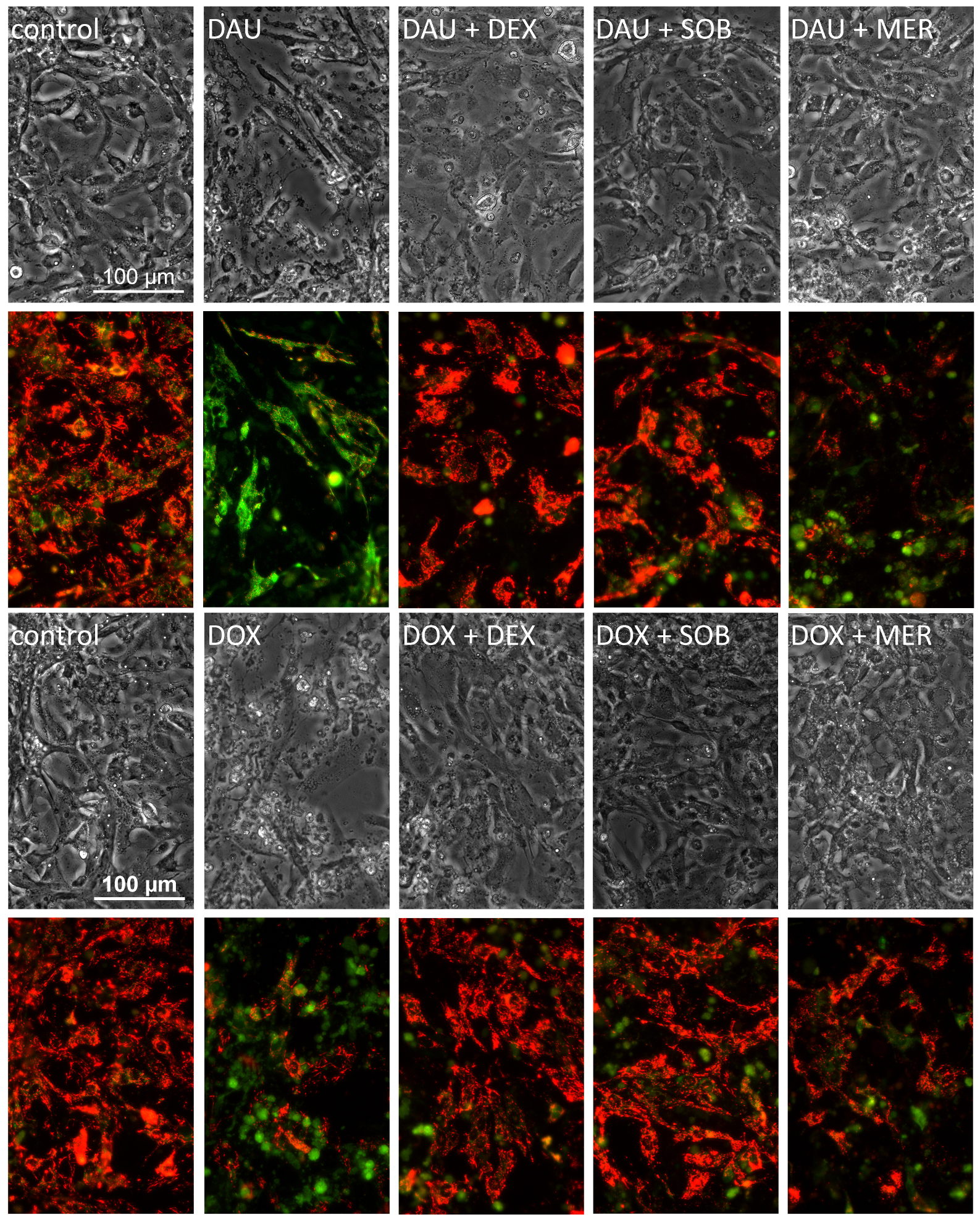

Supplement: Figure S1 — Effects of studied substances on cardiomyocyte morphology and mitochondrial depolarisation. Neonatal ventricular cardiomyocytes were pre-treated with 30 µM dexrazoxane (DEX), sobuzoxane (SOB) or merbarone (MER) for 3 h, then incubated with 1.2 µM of either daunorubicin (DAU) or doxorubicin (DOX) for 3 h, followed by an anthracycline-free incubation period for 48 h. Upper panels – brightfield phase contrast photomicrographs, lower panels – darkfield epifluorescence images of the same cells taken after loading with the JC-1 probe (red emission reflects mitochondrial inner membrane potential-dependent accumulation of probe dimers in actively respiring mitochondria, green fluorescence indicates monomers of the probe released into the cytoplasm after mitochondrial depolarisation, lack of fluorescence reflects probe release from necrotic or late-stage apoptotic cells). Scale bars represent 100 µm; all panels taken at the same magnification. (TIF) [file pone.0076676.s001.tif]

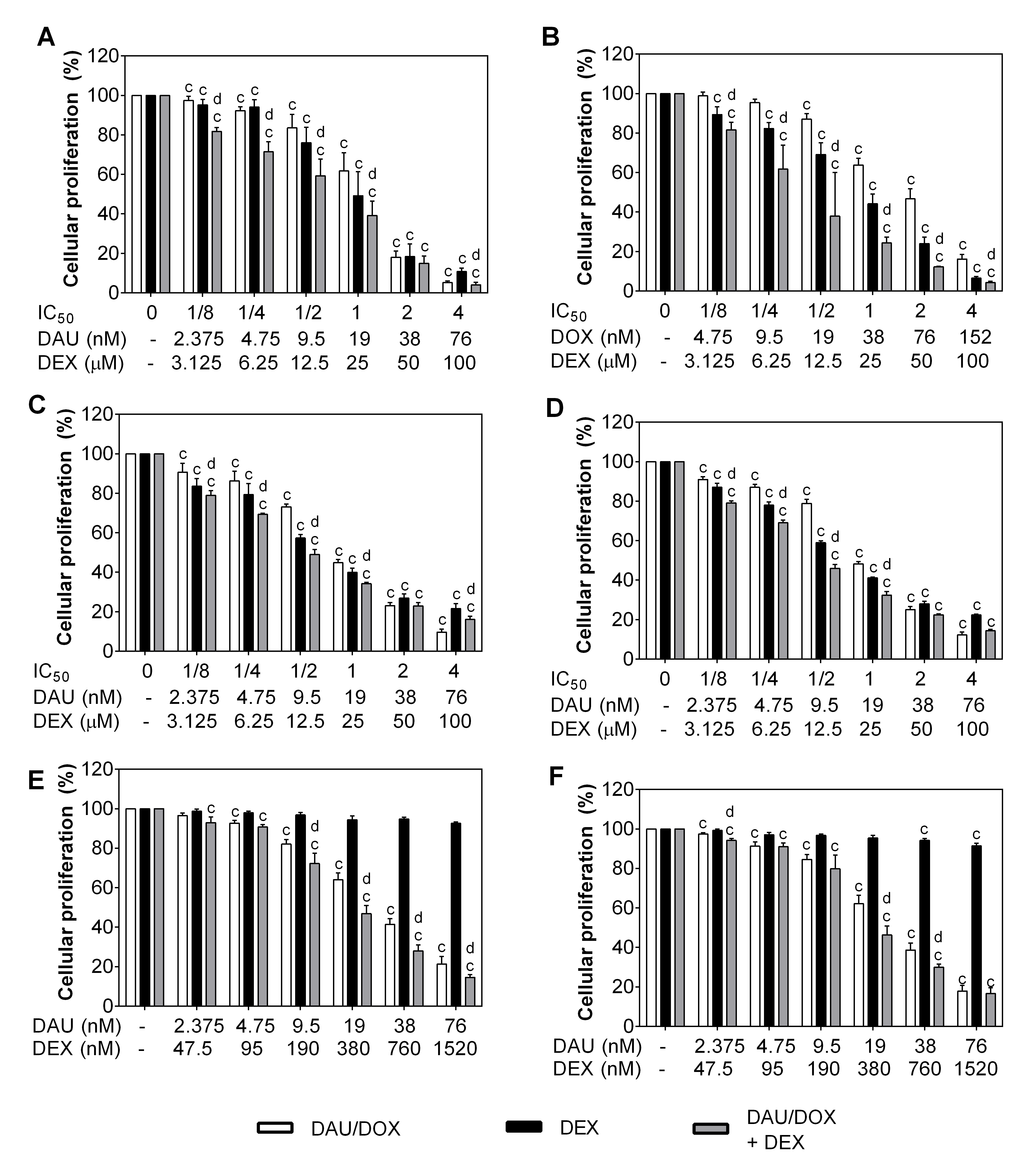

Supplement: Figure S2 — Effects of dexrazoxane and daunorubicin or doxorubicin on the proliferation of HL-60 cell line. Cells were incubated either continuously with dexrazoxane (DEX) and daunorubicin (DAU) or doxorubicin (DOX) for 72 h (A – B) or pre-incubated with DEX for 3 h (C) or 6 h (D) and then incubated for 72 h with all drugs at concentrations corresponding to their IC50 values and IC50 fractions and multiples (1/8; 1/4; 1/2; 1; 2; 4). Alternatively, cells were either co-incubated for 72 h (E) or pre-incubated with DEX for 3 h (F) and then co-incubated with DAU for 72 h at a ratio of 1:20 DAU:DEX. Data from 4 independent experiments expressed as the mean ± SD, statistical significance: c – compared to control; d – compared to DAU or DOX (one-way ANOVA with Dunnett’s post-test, P≤0.05). (TIF) [file pone.0076676.s002.tif]

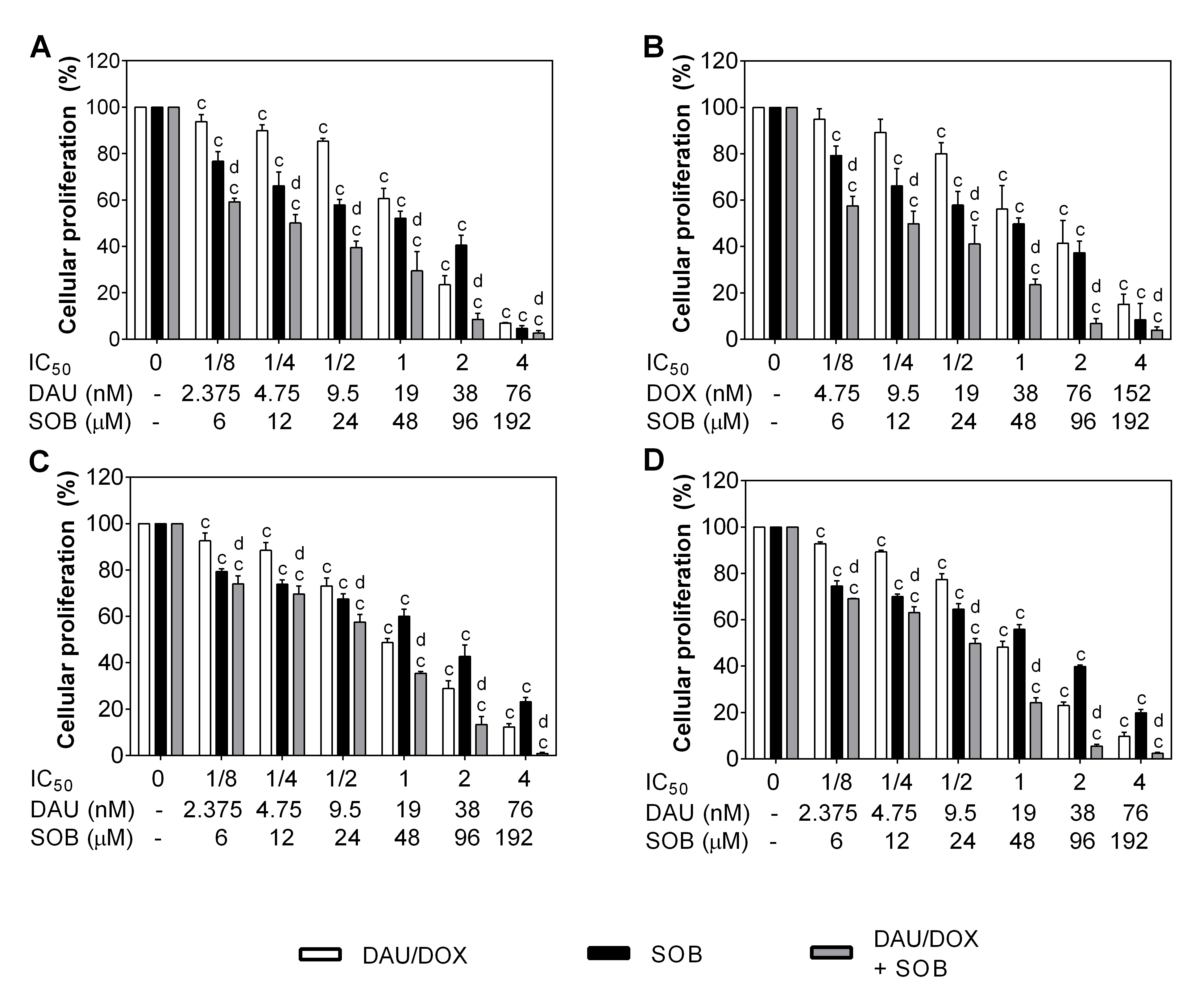

Supplement: Figure S3 — Effects of sobuzoxane and daunorubicin or doxorubicin on the proliferation of HL-60 cell line. Cells were incubated either continuously with sobuzoxane (SOB) and daunorubicin (DAU, A) or doxorubicin (DOX, B) for 72 h or pre-incubated with SOB for 3 h (C) or 6 h (D) and then incubated for 72 h with SOB and DAU at concentrations corresponding to their IC50 values and IC50 fractions and multiples (1/8; 1/4; 1/2; 1; 2; 4). Data from 4 independent experiments expressed as the mean ± SD, statistical significance: c – compared to control; d – compared to DAU or DOX (one-way ANOVA with Dunnett’s post-test, P≤0.05). (TIF) [file pone.0076676.s003.tif]

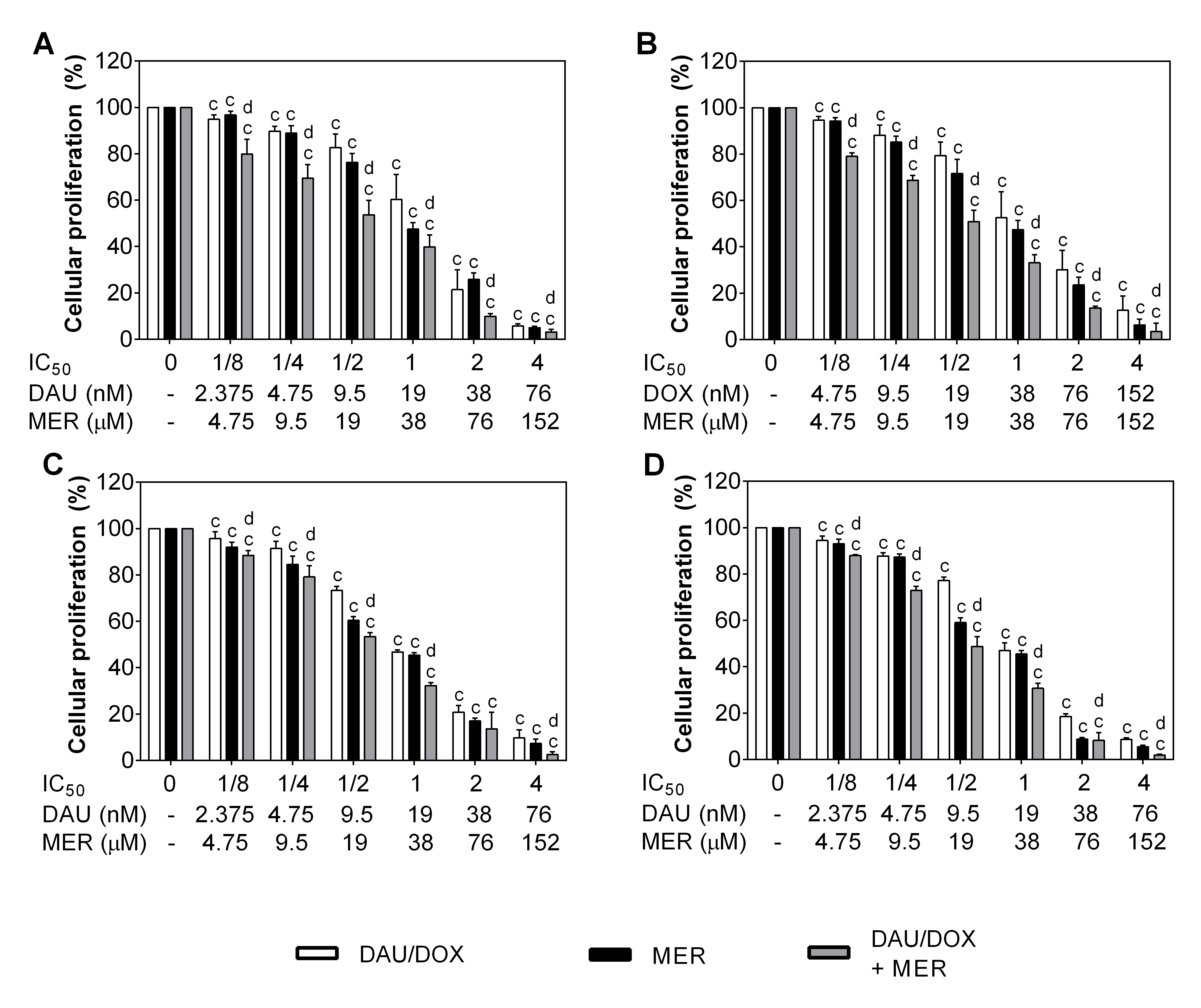

Supplement: Figure S4 — Effects of merbarone and daunorubicin or doxorubicin on the proliferation of HL-60 cell line. Cells were incubated either continuously with merbarone (MER) and daunorubicin (DAU, A) or doxorubicin (DOX, B) for 72 h or pre-incubated with MER for 3 h (C) or 6 h (D) and then incubated for 72 h with MER and DAU at concentrations corresponding to their IC50 values and IC50 fractions and multiples (1/8; 1/4; 1/2; 1; 2; 4). Data from 4 independent experiments expressed as the mean ± SD, statistical significance: c – compared to control; d – compared to DAU or DOX (one-way ANOVA with Dunnett’s post-test, P≤0.05). (TIF) [file pone.0076676.s004.tif]
