# Supplementary material for: Catalytic Inhibitors of Topoisomerase II Differently Modulate the Toxicity of Anthracyclines in Cardiac and Cancer Cells
Source: PLoS One. 2013 Oct 7;8(10):e76676. doi: 10.1371/journal.pone.0076676 (PMC3792022; doi:10.1371/journal.pone.0076676)
Supplement: Table S1 — The quantitative assessments of antiproliferative activities of combinations of doxorubicin (DOX) or daunorubicin (DAU) with dexrazoxane (DEX). The HL-60 cells were incubated with DEX without pre-incubation (DEX 0 h), or with 3-hour (DEX 3 h) or 6-hour pre-incubation (DEX 6 h) and then incubated with doxorubicin (DOX) or daunorubicin (DAU) in concentrations corresponding to their IC50 values and IC50 fractions and multiples (1/8; 1/4; 1/2; 1; 2; 4) or in a fixed 1:20 DAU:DEX concentration ratio. Values of combination indexes (CI) were calculated according to the method of Chou and Talalay as described in materials and methods using Calcusyn for Windows 2.0. CI < 1, ≈ 1 or > 1 means synergism, additive effect or antagonism, respectively. Data from four experiments are expressed as mean ± SD. (DOC) [file pone.0076676.s005.doc]

**Table S1. The quantitative assessments of antiproliferative activities of combinations of doxorubicin (DOX) or daunorubicin (DAU) with dexrazoxane (DEX).**

| IC_50_ multiples | DEX 0 h+ DOX | DEX 0 h+ DAU | DEX 3 h + DAU | DEX 6 h + DAU | DAU: DEX 1:20 0 h | DAU: DEX 1:20 3 h |
| --- | --- | --- | --- | --- | --- | --- |
| 1/8 | 0.177 ± 0.028 | 0.274 ± 0.034 | 0.390 ± 0.059 | 0.360 ± 0.019 | 0.581 ± 0.167 | 0.566 ± 0.070 |
| 1/4 | 0.215 ± 0.057 | 0.384 ± 0.118 | 0.540 ± 0.082 | 0.482 ± 0.030 | 0.893 ± 0.189 | 0.867 ± 0.140 |
| 1/2 | 0.327 ± 0.042 | 0.623 ± 0.127 | 0.577 ± 0.069 | 0.463 ± 0.037 | 0.683 ± 0.163 | 0.944 ± 0.243 |
| 1 | 0.349 ± 0.056 | 0.803 ± 0.140 | 0.725 ± 0.020 | 0.598 ± 0.039 | 0.599 ± 0.088 | 0.635 ± 0.075 |
| 2 | 0.453 ± 0.105 | 0.814 ± 0.196 | 0.972 ± 0.030 | 0.810 ± 0.043 | 0.650 ± 0.041 | 0.789 ± 0.057 |
| 4 | 0.503 ± 0.143 | 0.780 ± 0.234 | 1.404 ± 0.058 | 1.073 ± 0.074 | 0.712 ± 0.033 | 0.936 ± 0.116 |

The HL-60 cells were incubated with DEX without pre-incubation (DEX 0 h), or with 3-hour (DEX 3 h) or 6-hour pre-incubation (DEX 6 h) and then incubated with doxorubicin (DOX) or daunorubicin (DAU) in concentrations corresponding to their IC_50_ values and IC_50_ fractions and multiples (1/8; 1/4; 1/2; 1; 2; 4) or in a fixed 1:20 DAU:DEX concentration ratio. Values of combination indexes (*CI*) were calculated according to the method of Chou and Talalay as described in materials and methods using Calcusyn for Windows 2.0. *CI* < 1, ≈ 1 or > 1 means synergism, additive effect or antagonism, respectively. Data from four experiments are expressed as mean ± SD.
