# Supplementary material for: Catalytic Inhibitors of Topoisomerase II Differently Modulate the Toxicity of Anthracyclines in Cardiac and Cancer Cells
Source: PLoS One. 2013 Oct 7;8(10):e76676. doi: 10.1371/journal.pone.0076676 (PMC3792022; doi:10.1371/journal.pone.0076676)
Supplement: Table S2 — The quantitative assessments of antiproliferative activities of combinations of doxorubicin (DOX) or daunorubicin (DAU) with sobuzoxane (SOB). The HL-60 cells were incubated with SOB without pre-incubation (SOB 0 h), or with 3-hour (SOB 3 h) or 6-hour pre-incubation (SOB 6 h) and then incubated with doxorubicin (DOX) or daunorubicin (DAU) in concentrations corresponding to their IC50 values and IC50 fractions and multiples (1/8; 1/4; 1/2; 1; 2; 4). Values of combination indexes (CI) were calculated according to the method of Chou and Talalay as described in materials and methods using Calcusyn for Windows 2.0. CI < 1, ≈ 1 or > 1 means synergism, additive effect or antagonism, respectively. Data from four experiments are expressed as mean ± SD. (DOC) [file pone.0076676.s006.doc]

**Table S2. The quantitative assessments of antiproliferative activities of combinations of doxorubicin (DOX) or daunorubicin (DAU) with sobuzoxane (SOB).**

| IC_50_ multiples | SOB 0 h + DOX | SOB 0 h + DAU | SOB 3 h + DAU | SOB 6 h + DAU |
| --- | --- | --- | --- | --- |
| 1/8 | 0.123 ± 0.016 | 0.154 ± 0.017 | 0.277 ± 0.023 | 0.232 ± 0.016 |
| 1/4 | 0.194 ± 0.017 | 0.239 ± 0.008 | 0.471 ± 0.054 | 0.387 ± 0.050 |
| 1/2 | 0.296 ± 0.035 | 0.360 ± 0.018 | 0.636 ± 0.033 | 0.530 ± 0.029 |
| 1 | 0.333 ± 0.096 | 0.534 ± 0.108 | 0.654 ± 0.068 | 0.487 ± 0.081 |
| 2 | 0.249 ± 0.133 | 0.399 ± 0.121 | 0.501 ± 0.099 | 0.298 ± 0.049 |
| 4 | 0.331 ± 0.192 | 0.357 ± 0.120 | 0.118 ± 0.041 | 0.324 ± 0.047 |

The HL-60 cells were incubated with SOB without pre-incubation (SOB 0 h), or with 3-hour (SOB 3 h) or 6-hour pre-incubation (SOB 6 h) and then incubated with doxorubicin (DOX) or daunorubicin (DAU) in concentrations corresponding to their IC_50_ values and IC_50_ fractions and multiples (1/8; 1/4; 1/2; 1; 2; 4). Values of combination indexes (*CI*) were calculated according to the method of Chou and Talalay as described in materials and methods using Calcusyn for Windows 2.0. *CI* < 1, ≈ 1 or > 1 means synergism, additive effect or antagonism, respectively. Data from four experiments are expressed as mean ± SD.
