# Supplementary material for: Catalytic Inhibitors of Topoisomerase II Differently Modulate the Toxicity of Anthracyclines in Cardiac and Cancer Cells
Source: PLoS One. 2013 Oct 7;8(10):e76676. doi: 10.1371/journal.pone.0076676 (PMC3792022; doi:10.1371/journal.pone.0076676)
Supplement: Table S3 — The quantitative assessments of antiproliferative activities of combinations of doxorubicin (DOX) or daunorubicin (DAU) with merbarone (MER). The HL-60 cells were incubated with MER without pre-incubation (MER 0 h), or with 3-hour (MER 3 h) or 6-hour pre-incubation (MER 6 h) and then incubated with doxorubicin (DOX) or daunorubicin (DAU) in concentrations corresponding to their IC50 values and IC50 fractions and multiples (1/8; 1/4; 1/2; 1; 2; 4). Values of combination indexes (CI) were calculated according to the method of Chou and Talalay as described in materials and methods using Calcusyn for Windows 2.0. CI < 1, ≈ 1 or > 1 means synergism, additive effect or antagonism, respectively. Data from four experiments are expressed as mean ± SD. (DOC) [file pone.0076676.s007.doc]

**Table S3. The quantitative assessments of antiproliferative activities of combinations of doxorubicin (DOX) or daunorubicin (DAU) with merbarone (MER).**

| IC_50_ multiples | MER 0 h + DOX | MER 0 h + DAU | MER 3 h + DAU | MER 6 h + DAU |
| --- | --- | --- | --- | --- |
| 1/8 | 0.303 ± 0.075 | 0.308 ± 0.049 | 0.440 ± 0.088 | 0.505 ± 0.059 |
| 1/4 | 0.418 ± 0.111 | 0.430 ± 0.042 | 0.588 ± 0.119 | 0.533 ± 0.029 |
| 1/2 | 0.486 ± 0.099 | 0.570 ± 0.031 | 0.571 ± 0.077 | 0.546 ± 0.048 |
| 1 | 0.571 ± 0.103 | 0.816 ± 0.065 | 0.674 ± 0.075 | 0.670 ± 0.035 |
| 2 | 0.519 ± 0.143 | 0.563 ± 0.096 | 0.669 ± 0.205 | 0.475 ± 0.135 |
| 4 | 0.314 ± 0.100 | 0.563 ± 0.230 | 0.476 ± 0.124 | 0.361 ± 0.044 |

The HL-60 cells were incubated with MER without pre-incubation (MER 0 h), or with 3-hour (MER 3 h) or 6-hour pre-incubation (MER 6 h) and then incubated with doxorubicin (DOX) or daunorubicin (DAU) in concentrations corresponding to their IC_50_ values and IC_50_ fractions and multiples (1/8; 1/4; 1/2; 1; 2; 4). Values of combination indexes (*CI*) were calculated according to the method of Chou and Talalay as described in materials and methods using Calcusyn for Windows 2.0. *CI* < 1, ≈ 1 or > 1 means synergism, additive effect or antagonism, respectively. Data from four experiments are expressed as mean ± SD.
